# A Welcomed Death

**Published:** 1859-08

**Authors:** 


### A WELCOMED DEATH.

As the Fourth Avenue cars were carrying their four o'clock freight from Wall Street to the "regal region" about Union Square and Irving Place, a stranger next us, inquired with a foreign accent, the meaning of a funeral procession just passing; "Death of Humboldt," replied a citizen. "Death of Humbug," soliloquized the stranger. "Humbug dead in New York—Glory, Halleluyer!" But as if the news were too good to be true, and to assure himself, he made up to the keenest looking man in the car, and with evident interest and sincerity inquired, "Is that Humbug—Humbug dead?" "Yes," said Jacob Little, (the gentleman addressed,) in a commisserating tone, with a look out of that twinkler not to match, "Dead!"

